# Effect of *n*-octanol on impurity removal by reverse flotation of magnesite ore

**DOI:** 10.1038/s41598-022-19377-0

**Published:** 2022-09-02

**Authors:** Pengcheng Li, Xiaoan Li, Shujuan Dai, Wenhan Sun, Bin Zhou

**Affiliations:** 1 School of Chemical Engineering, University of Science and Technology Liaoning, Anshan, 114051 China; 2 School of Resources and Civil Engineering, Northeastern University, Shenyang, 110819 China; 3 Haicheng Huayu Refractory Material Co., Ltd., Anshan, 114206 China

**Keywords:** Solid Earth sciences, Mineralogy

## Abstract

Dodecylamine is one of the most commonly used amine collectors for the reverse flotation of magnesite ore. Through a combination of experimental research and computational simulation, the effect of *n*-octanol on the removal of impurities by the reverse flotation of magnesite ore was studied. The test results show that when the dosage of dodecylamine was 60 mg/L, the flotation rates of magnesite and dolomite were 59.53% and 58.02%, respectively, and the flotation rate of quartz was 97.60%. In the presence of *n*-octanol, the flotation rate of magnesite decreased to 56.41%, and the flotation rates of dolomite and quartz increased to 61.30% and 99.59%, respectively. The test results show that the addition of *n*-octanol can improve the selectivity of minerals under the same amount of collector. The adsorption of dodecylamine (dodecylamine and *n*-octanol) on the surface of magnesite, dolomite and quartz was simulated using quantum chemical calculations based on density functional theory (DFT) and implemented in the CASTEP module of Materials Studio. The results show that dodecylamine can adsorb to magnesite, dolomite and quartz, and the adsorption effect was strongest on quartz. After adding *n*-octanol, the population value of the bond between the agent and magnesite decreased from 0.19 to 0.17, indicating that the adsorption effect of the agent on magnesite was weakened. The population value of the bond between the drug and dolomite increased from 0.19 to 0.23, indicating that the adsorption effect of the drug on dolomite was enhanced. H28, H29, and H2 in the drug form bonds with O12, O20, and O20 on the surface of quartz (101), respectively, and the population values were 0.43, 0.25, and 0.09, respectively. The adsorption sites of the drug and the quartz were increased, and the adsorption effect of the quartz was markedly improved. The test and simulation results show that the dosage of the agent can be reduced in the presence of *n*-octanol. *N*-octanol is not only beneficial to the removal of silicon by amine reverse flotation but also has a certain beneficial effect on the removal of calcium by reverse flotation.

## Introduction

Magnesite is widely used in refractories due to its high fire resistance and cohesiveness^1,2^. In recent years, due to the overexploitation of high-grade magnesite ore, the availability of high-quality magnesite resources has decreased, and strategies to use low-grade magnesite resources have become particularly important^3,4^. The useful mineral in magnesite ore is magnesite, and the gangue minerals are mainly silica-containing minerals and dolomite-based calcium-containing minerals. Compared with other methods, the flotation method is considered to be a more effective method for treating low-grade magnesite^5–8^. At present, the purification of magnesite is mainly based on reverse flotation. The use of single cationic collector reverse flotation can effectively remove silicon-containing impurity minerals in magnesite ore. The calcium-bearing gangue minerals dolomite and magnesite have similar floatability and are difficult to separate. In this study, single cation collector (adding *n*-octanol) reverse flotation could remove a small amount of calcium while removing silicon.

The widely used collectors for magnesite flotation are amine collectors^4,9^ and sodium oleate collectors^8^. Amine collectors (such as dodecylamine and etheramine) are widely used in the reverse flotation of magnesite^10^. Some researchers modified fatty amines, resulting in collectors like quaternary ammonium surfactants and gemini surfactants^11–14^. However, most cationic surfactants have the disadvantages of high cost and poor selectivity^15^. Many researchers^16–19^ have proven that adding nonionic surfactants (such as fatty alcohols) to cationic collectors can reduce the amount of collectors and improve silicate cation flotation. Filippov et al.^20^ showed that the combination of Cataflot (primary amine) and PX4826 (nonionic reagent) can improve the floatability of silicates and calcium-containing minerals while reducing amine consumption. Vidyadhar et al.^18,19^ found that the coadsorption of long-chain alcohols and amine cations resulted in the formation of a closed filling surface layer, confirming that the synergy of amine adsorption increased or decreased in the presence of alcohol.

Adding alcohol to amine collectors can improve the selectivity of flotation and the recovery of silicate, and the influence of alcohol structure on flotation has been widely studied. However, relatively few studies have examined the use of quantum mechanics to calculate the impurity removal from magnesite in the presence of alcohol. This study combined experimental research and computational simulation to show that adding *n*-octanol can remove a small amount of calcium while removing silicon by reverse flotation. The results help to elucidate the synergistic adsorption mechanism of dodecylamine and *n*-octanol on mineral surfaces.

## Test materials and methods

### Test materials

#### Test sample

**Figure 1:**
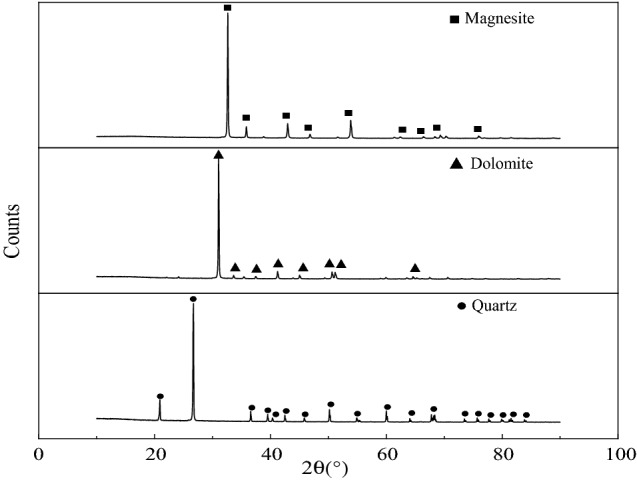
XRD pattern of the sample.

The pure minerals magnesite, dolomite, and quartz were manually selected, crushed, sieved, and ground through a series of processes, and a − 0.074 mm particle size was finally obtained for use. Quartz single minerals needed to be soaked in dilute hydrochloric acid, repeatedly washed with distilled water to stabilize the pH value of the solution at approximately 7, and set aside after drying. The chemical element analysis of magnesite, dolomite, and quartz single minerals is shown in Table 1, and the X-ray diffraction test results are shown in Fig. 1.

**Table 1:** Chemical composition analysis of magnesite, dolomite and quartz (%).

Sample	CO_2_	CaO	Fe_2_O_3_	Al_2_O_3_	SiO_2_	MgO	Other	MgO (IL = 0)
Magnesite	51.81	0.21	0.25	0	0.09	47.64	–	98.86
Dolomite	45.78	30.32	0.09	0.04	0.36	23.41	–	–
Quartz	–	–	0.007	–	99.80	–	0.193	–

Tables 1, 2 and Fig. 1 show that the impurity content in pure magnesite mineral samples was relatively small, of which the MgO content was 47.64%. When the burning reduction was 0, the MgO content was 98.86%, and the magnesite content exceeded 97%. The main component of dolomite minerals was dolomite, and the content of impurity elements, such as iron, silicon, and aluminium, was low; the main component of pure quartz minerals was SiO_2_, with a content of 99.80%. Three types of minerals met the requirements of the pure mineral flotation test.

**Table 2:** Adsorption energy of adsorbate on the surface of magnesite (101), dolomite (101) and quartz (101).

Mineral	Adsorbate	
	DDA	NDA
	Adsorption energy (kJ/mol)	
Magnesite	− 197.14	− 330.90
Dolomite	− 127.96	− 206.66
Quartz	− 464.13	− 573.14

#### Test agent

The reagents used in the flotation test were dodecylamine, *n*-octanol and hydrochloric acid, which were chemically pure or analytically pure and purchased from the reagent sales company.

### Test method

#### Single mineral flotation test

The pure mineral flotation test utilized a hanging tank XFGC-type flotation machine used in the laboratory. The volume of the flotation tank was 30 mL, the speed of the flotation machine was 1800 r/min, and the test water was distilled water. Three grams of pure minerals and 25 mL of distilled water were added each time. After adding the regulator and collector (collector + *n*-octanol), the mixture was stirred for 3 min. After flotation for 4 min, a circulating water vacuum pump was used to filter, dry, and weigh the sample.

#### Quantum mechanics calculation simulation

The calculation simulation utilized the CASTEP (Cambridge Serial Total Energy Package) quantum mechanics module in Materials Studio (hereinafter referred to as MS). This module uses the density functional method for simulation. The convergence criteria of the ideal structure and for the energy calculation were as follows: (a) an energy tolerance of 2 × 10^−5^ eV/atom, (b) a maximum-force tolerance of 0.05 eV/Å, (c) a maximum-displacement tolerance of 0.002 Å and (d) a self-consistent-field tolerance of 1.0 × 10^−6^ eV/atom.

Establishing a stable surface structure and relaxing the surface atoms is an important step before the interaction between the agent and the mineral crystal. In this paper, through the relaxation of different cleavage surfaces of magnesite, dolomite and quartz, the surface energy of each cleavage surface was compared to determine the best surface for the agent and minerals. The surface energy was calculated as follows^21,22^:

1 $${\text{E}}_{{{\text{surf}}}} = [ {{\text{E}}_{{{\text{slab}}}} - ( {{\text{N}}_{{{\text{slab}}}}/{\text{N}}_{{{\text{bulk}}}} } )\cdot{\text{E}}_{{{\text{bulk}}}} } ]/{\text{2A}}$$

where E_slab_ is the energy of the surface slab, E_bulk_ is the single-cell volume energy, N_slab_ is the number of atoms in the layer, N_bulk_ is the number of atoms contained in a unit-cell volume, A is the specific surface area, and the factor 1/2 derives from the two faces of the *z*-axis in the surface layer.

The stability of the adsorption system was represented by the adsorption energy of the agent on the surface of the mineral. The total adsorption energy was^23,24^:

2 $$\Delta {\text{E}}_{{{\text{ads}}}} = {\text{ E}}_{{{\text{complex}}}} - ( {{\text{E}}_{{{\text{adsorbate}}}} + {\text{ E}}_{{{\text{mineral}}}} } )$$

where E_complex_ is the total energy of the optimal adsorbate and mineral structure, E_adsorbate_ is the total energy of the adsorbate, and E_mineral_ is the energy required to cut the mineral surface. A negative ΔE_ads_ indicates exothermic adsorption, i.e., the adsorbate has an increasingly stronger effect on the mineral surface.

When ΔE_ads_ was negative, the reaction was exothermic, and the magnitude of ΔE_ads_ directly correlated with the strength and quality of the effect of the agent on the mineral surface, indicating that the adsorption process is more likely to occur.

## Test results and discussion

### Experimental research

**Figure 2:**
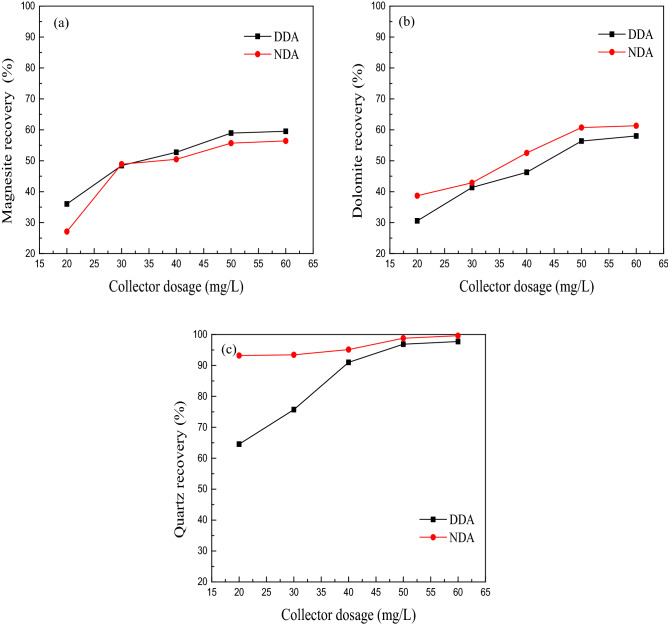
The effect of *n*-octanol on the flotation of magnesite (**a**), dolomite (**b**) and quartz (**c**) with dodecylamine (*DDA* dodecylamine, *NDA* dodecylamine + *n*-octanol).

The pulp pH was adjusted to 5–6 with hydrochloric acid. The effect of the amount of collector DDA on the floatability of magnesite, dolomite and quartz under the condition of *n*-octanol (the mass ratio of dodecylamine to *n*-octanol is 4:1) was investigated. The results are shown in Fig. 2.

The test results in Fig. 2a show that the rate of magnesite buoyancy increased from 36.02 to 52.75% as the dosage of DDA increased from 20 to 40 mg/L. Then, as the amount of the collector increased, the buoyancy rate of magnesite increased. Slightly increasing the DDA dosage from 40 to 60 mg/L increased the magnesite buoyancy rate from 52.75 to 59.53%. After adding *n*-octanol, the floating rate of magnesite decreased. The decrease was most pronounced (8.92%) when the dosage of DDA was 20 mg/L. When the dosage of DDA exceeded 40 mg/L, the decrease ranged from 2–3.5%.

The test results in Fig. 2b show that the dosage of DDA increased from 20 to 50 mg/L, and the floating rate of dolomite increased from 30.57 to 56.37%. As the amount of collector continued to increase, the buoyancy rate of dolomite also continued to increase, but only slightly. After adding *n*-octanol, the floating rate of dolomite increased from 1 to 8%, which is beneficial to improve the effect of reverse flotation decalcification.

The test results in Fig. 2c show that the recovery of quartz was greatly increased when the amount of collector was 20–40 mg/L. When 40 mg/L DDA was added, the flotation rate of quartz was 90.99%. The amount of collector was 40–60 mg/L. As the amount of collector increased, the recovery of quartz slowed. When the amount of collector was 50 mg/L, the buoyancy rate of quartz increased to 96.85%; when the amount of collector was 60 mg/L, the buoyancy rate of quartz reached 97.60%. After adding *n*-octanol, the floating rate of quartz significantly increased. When the amount of collector was 20 mg/L, the floating rate of quartz exceeded 90%. When the amount of collector was 50 mg/L, the increase was 1.94%.

The above test results show that continuously increasing the amount of collectors allows DDA to increase the buoyancy rate of magnesite, dolomite and quartz, but this improvement was limited for magnesite and dolomite. When the dosage was less than 60 mg/L, the flotation rate of magnesite and dolomite did not exceed 60%. DDA has a stronger ability to collect quartz. When the dosage exceeded 50 mg/L, the recovery rate of quartz exceeded 95%. After adding *n*-octanol, the floating rate of magnesite decreased. The decrease was most marked (8.92%) when the dosage of DDA was 20 mg/L. The floating rate of dolomite increased from 1 to 8%, which is beneficial to improve the effect of reverse flotation decalcification. The floating rate of quartz was clearly evident, and the floating rate of quartz exceeded values of 90% for a lower amount of collector. In summary, in the presence of *n*-octanol, the amount of drug used can be reduced. When reverse flotation magnesite ore removes silicon, it can remove part of the calcium and improve the purity of the magnesite.

### Calculation and analysis

#### The construction of magnesite (101), dolomite (101) and quartz (101) surfaces

Surface energy is generally defined as the reversible work required to produce a unit surface area. Lower value indicates a more stable surface and a more accurate surface structure^25^. The thickness of the magnesite atomic layer ranged from 4.129 to 23.740 Å, the thickness of the dolomite atomic layer ranged from 4.351 to 24.625 Å, the thickness of the atomic layer ranged from 2.535 to 19.399 Å, and the vacuum layers of the three minerals ranged in thickness from 40 to 65 Å. The surface energy calculation formula is shown in (1).

**Figure 3:**
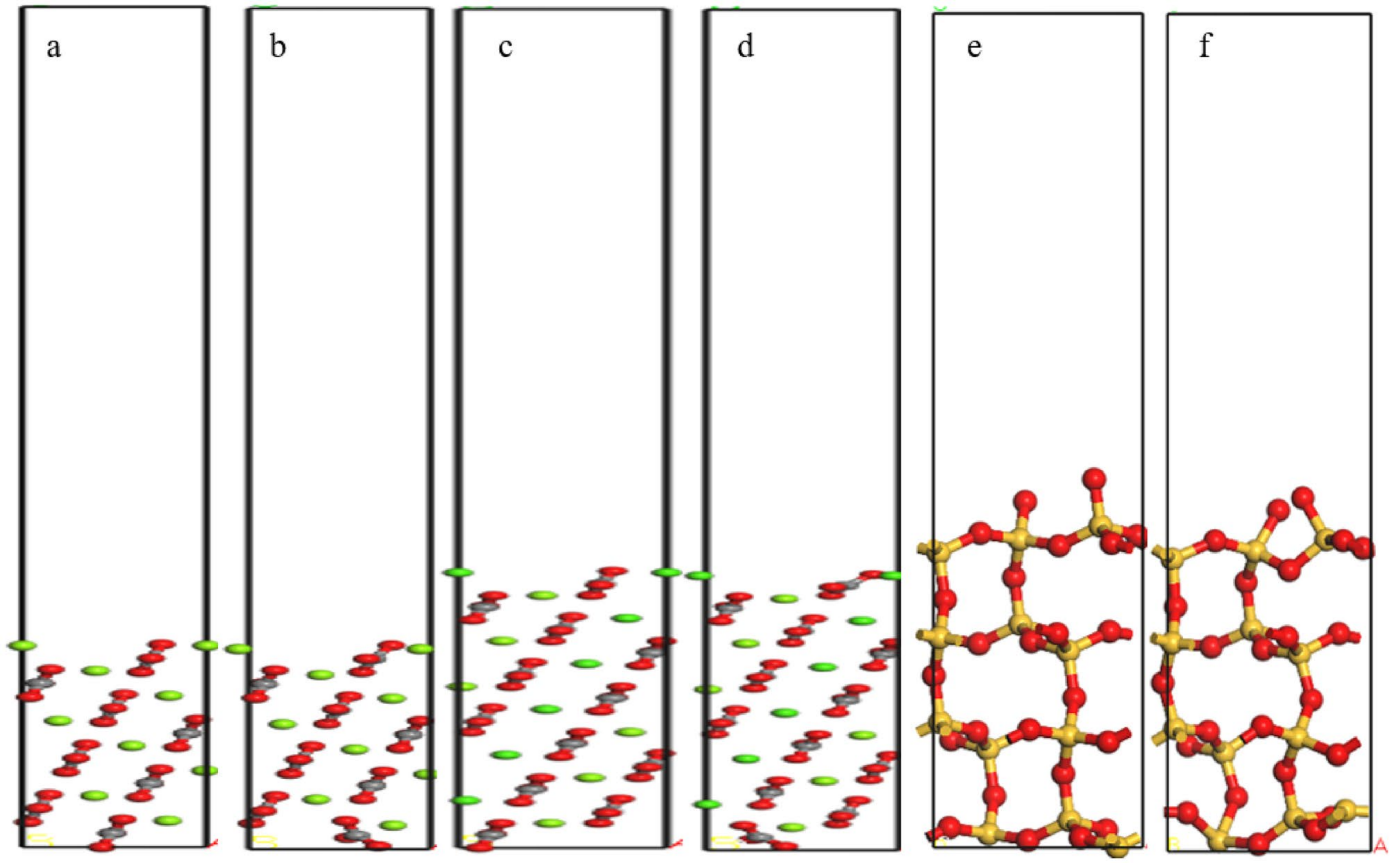
Unit cell model of the magnesite (101) (**a**,**b**), dolomite (101) (**c**,**d**) and quartz (101) (**e**,**f**) surfaces before relaxation (**a**,**c**,**e**) and after relaxation (**b**,**d**,**f**).

The variation range of the surface energy was less than 0.05 J/m^2^, indicating that it had stabilized. For subsequent calculations, the atomic layer thickness of magnesite was 12.654 Å, the vacuum layer thickness was 50 Å, and the surface energy at this time was 0.803 J/m^2^. Dolomite was selected to have an atomic layer thickness of 24.625 Å and a vacuum layer thickness of 50 Å, with a surface energy of 0.712 J/m^2^. The selected atomic layer thickness of quartz was 12.654 Å, the vacuum layer thickness was 50 Å, and the surface energy at this time was 1.318 J/m^2^. The surface models of the three minerals before and after relaxation are shown in Fig. 3, and the model after relaxation was used as the subsequent adsorption model.

#### Adsorption of the agent on the surface of magnesite (101), dolomite (101) and quartz (101)

CASTEP was used to simulate and calculate the adsorption energy of the agent on the surface of magnesite (101), dolomite (101) and quartz (101). A negative value of adsorption energy indicates exothermic adsorption, i.e., the adsorbate has a better and stronger effect on the surface of magnesite. The formula for the total adsorption energy is shown in (2).

**Figure 4:**
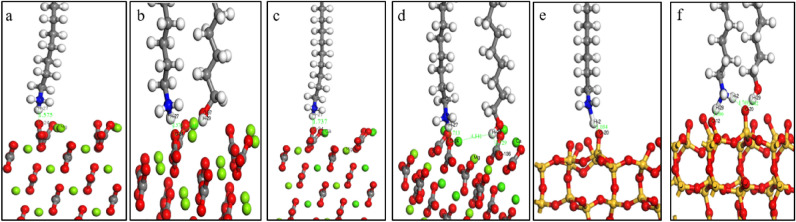
Geometric adsorption structure of DDA (**a**,**c**,**e**) and NDA (**b**,**d**,**f**) on the surface of magnesite (101) (**a**,**b**), dolomite (101) (**c**,**d**) and quartz (101) (**e**,**f**).

Before adsorption, DDA and NDA were placed in a 20 × 20 × 20 cubic unit cell for optimization. The k-point was chosen as the gamma point. The optimized geometric adsorption structures of DDA and NDA on the surface of magnesite, dolomite and quartz are shown in Fig. 4, and the calculated related adsorption energies are shown in Table 2.

Figure 4a,b and Table 2 show that the adsorption energy of DDA on the surface of magnesite (101) was − 197.14 kJ/mol, and the bond length of H27 and O24 was 1.575 Å. The adsorption energy of NDA on the surface of magnesite (101) was − 330.90 kJ/mol, and the bond length of H27 and O24 was 1.570 Å. DDA and NDA can be adsorbed on the surface of magnesite, and the effect of NDA on the surface of magnesite (101) was stronger than that of DDA. However, judging from the bond length, the two functions are essentially the same, perhaps because the addition of *n*-octanol increases the hydrogen bond on the surface of magnesite, resulting in a decrease in adsorption energy.

Figure 4c,d and Table 2 show that the adsorption energy of DDA on the surface of dolomite (101) was − 127.96 kJ/mol, and the bond length of H27 and O34 was 1.737 Å. The adsorption energy of NDA on the surface of dolomite (101) was − 206.66 kJ/mol, and the bond length of H27 and O34 was 1.713 Å. DDA and NDA could adsorb to the surface of dolomite. The adsorption energy and bond length show that NDA has stronger effects on the surface of dolomite (101) than DDA, perhaps because the addition of *n*-octanol increased the hydrogen bond on the surface of dolomite, resulting in a decrease in adsorption energy.

Figure 4e,f and Table 2 show that the adsorption energy of DDA on the surface of quartz (101) was − 464.13 kJ/mol, and the bond length of H2 and O20 was 1.034 Å. The adsorption energy of NDA on the surface of quartz (101) was − 573.14 kJ/mol. The bond length of H28 and O12 was 1.090 Å, the bond length of H29 and O20 was 1.362 Å, and the bond length of H2 and O20 was 1.769 Å. This finding shows that DDA and NDA could adsorb to the surface of quartz. The adsorption energy and bond length show that NDA has stronger effects on the surface of quartz (101) than DDA, perhaps because the addition of *n*-octanol increases the adsorption sites on the quartz surface, increases the hydrogen bond on the quartz surface, and reduces the adsorption energy. To further illustrate the problem, the electronic structures of the three minerals before and after interaction with the agent were analysed.

#### Analysis of the electronic structure of magnesite, dolomite and quartz

The adsorption of DDA and NDA to the surface of magnesite (101) occurred between H27 and O24. The adsorption of DDA and NDA to the surface of dolomite (101) occurred between H27 and O34. The adsorption of DDA to the surface of quartz (101) occurred between H2 and O20, and the adsorption of NDA occurred between H28 and O12, H29 and O20, and H2 and O20 to the surface of quartz (101). The specific results are shown in Tables 3 and 4.

**Table 3:** DDA Mulliken population analysis on the surface of Magnesite (101), dolomite (101) and quartz (101).

Mineral	Atom		*s*	*p*	Total (e)	Charge (e)
Magnesite	H27	Before adsorption	0.64	–	0.64	0.36
		After adsorption	0.60	–	0.60	0.40
	O24	Before adsorption	1.84	4.90	6.74	− 0.74
		After adsorption	1.84	4.87	6.70	− 0.70
Dolomite	H27	Before adsorption	0.94	–	0.94	0.06
		After adsorption	0.66	–	0.66	0.34
	O34	Before adsorption	1.93	1.42	3.35	2.65
		After adsorption	1.85	3.96	5.80	0.20
Quartz	H2	Before adsorption	0.56	–	0.56	0.44
		After adsorption	0.54	–	0.54	0.46
	O20	Before adsorption	1.89	4.79	6.68	− 0.68
		After adsorption	1.82	5.27	7.09	− 1.09

**Table 4:** NDA Mulliken population analysis on the surface of magnesite (101), dolomite (101) and quartz (101).

Mineral	Atom		*s*	*p*	Total(e)	Charge(e)
Magnesite	H27	Before adsorption	0.60	–	0.60	0.40
		After adsorption	0.61	–	0.61	0.39
	O24	Before adsorption	1.84	4.88	6.72	− 0.72
		After adsorption	1.83	4.90	6.73	− 0.73
Dolomite	H27	Before adsorption	0.96	–	0.96	0.04
		After adsorption	1.18	–	1.18	− 0.18
	O34	Before adsorption	1.94	1.66	3.60	2.40
		After adsorption	1.92	1.66	3.58	2.42
Quartz	H28	Before adsorption	0.55	–	0.55	0.45
		After adsorption	0.59	–	0.59	0.41
	O12	Before adsorption	1.90	4.82	6.72	− 0.72
		After adsorption	1.85	5.21	7.06	− 1.06
	H2	Before adsorption	0.54	–	0.54	0.45
		After adsorption	0.59	–	0.59	0.41
	H29	Before adsorption	0.60	–	0.60	0.40
		After adsorption	0.54	–	0.54	0.46
	O20	Before adsorption	1.89	4.83	6.72	− 0.72
		After adsorption	1.87	5.21	7.08	− 1.08

Table 3 shows that after DDA adsorbed to the surface of magnesite (101), the electrons of the 1 s orbital of the H27 atom reduced from 0.64e to 0.60e, and the total electrons reduced from 0.64e to 0.60e, a decrease of 0.04e. The hydrogen band was + 0.40e. The 2 s orbital of the O24 atom did not change, whereas the 2p orbital dropped from 4.90e to 4.87e, and the total electrons dropped from 6.74e to 6.70e, a decrease of 0.04e; the oxygen band was − 0.70e, which indicates that DDA is in magnesite. The surface could be electrostatically adsorbed. Table 4 shows that after NDA adsorbed to the surface of magnesite (101), the electrons of the 1 s orbital of the H27 atom increased from 0.60e to 0.61e, and the total electrons increased from 0.60e to 0.61e. The hydrogen band was at + 0.39e. The orbital of the O24 atom decreased from 1.84e to 1.83e, the 2p orbital increased from 4.88e to 4.90e, and the total electrons increased from 6.72e to 6.73e, an increase of 0.01e; the oxygen band was at − 0.73e. This finding shows that NDA could electrostatically adsorb to the surface of magnesite.

Table 3 shows that after DDA adsorbed to the surface of dolomite (101), the electrons of the 1 s orbital of the H27 atom reduced from 0.94e to 0.66e, and the total electrons reduced from 0.84e to 0.66e, a decrease of 0.28e. The hydrogen atom band was at + 0.34e. The 2 s orbital of the O34 atom decreased from 1.93e to 1.85e, and the 2p orbital increased from 1.42e to 3.96e. The total electrons increased from 3.35e to 5.80e, an increase of 2.45e, and the oxygen band was at + 0.20e. This finding shows that DDA could electrostatically adsorb to the surface of dolomite, and some of the electrons of hydrogen atoms on DDA were transferred to oxygen atoms on the surface of dolomite. Table 4 shows that after NDA adsorbed to the surface of dolomite (101), the electrons of the 1 s orbital of the H27 atom increased from 0.96e to 1.18e, an increase of 0.22e. The hydrogen band was at − 0.18e. The 2 s orbital of the O34 atom decreased from 1.94e to 1.92e, whereas the 2p orbital remained unchanged. The total electrons decreased from 3.60e to 3.58e, a decrease of 0.02e, and the oxygen band was at + 2.42e. This finding indicates that NDA could electrostatically adsorb to the surface of dolomite, and some of the electrons of oxygen atoms on the surface of dolomite transferred to the hydrogen atoms of DDA.

Table 3 shows that after DDA adsorbed to the surface of quartz (101), the total electrons of H2 atoms reduced from 0.56e to 0.54e, and the hydrogen atom band was at + 0.46e. The 2 s orbital of the O20 atom decreased from 1.89e to 1.82e, whereas the 2p orbital increased from 4.79e to 5.27e, and the total electrons increased from 6.68e to 7.09e. The oxygen band was at − 1.09e. This finding shows that DDA could electrostatically adsorb to the surface of quartz, and some of the electrons of hydrogen atoms on DDA transferred to oxygen atoms on the surface of quartz. Figure 4f and Table 4 show that after NDA adsorbed to the surface of quartz (101), one more O12 adsorption site existed on the surface of quartz. The total electrons of the H28 atom increased from 0.55e to 0.59e, and the hydrogen atom band was at + 0.41e. The 2 s orbital of the O12 atom decreased from 1.90e to 1.85e, whereas the 2p orbital increased from 4.82e to 5.21e, and the total electrons increased from 6.72e to 7.06e. The oxygen band was at − 1.06e. The total electrons of H2 atoms increased from 0.54e to 0.59e, and the hydrogen atom band was at + 0.41e. The total electrons of the H29 atom reduced from 0.60e to 0.54e, and the hydrogen atom band was at + 0.46e. The 2 s orbital of the O20 atom decreased from 1.89e to 1.87e, the 2p orbital increased from 4.83e to 5.21e, and the total electrons increased from 6.72e to 7.08e. The oxygen band was at − 1.08e. This finding shows that NDA could electrostatically adsorb to the surface of quartz.

The strength of the bond can be expressed by the relative size of Mulliken's overlapping population. A positive overlap population (the electron cloud between two atoms overlaps) indicates a bonding state. The population analysis of DDA and NDA adsorbed to the surface of magnesite (101), dolomite (101) and quartz (101) is shown in Table 5.

**Table 5:** Bond and population analyses of DDA and NDA adsorbed to magnesite (101), dolomite (101) and quartz (101) surfaces.

Mineral	Adsorbate	Bond	Population	Bond length(Å)
Magnesite	DDA	H27–O24	0.19	1.575
	NDA	H27–O24	0.17	1.570
Dolomite	DDA	H27–O34	0.19	1.737
	NDA	H27–O34	0.23	1.713
Quartz	DDA	H2–O20	0.47	1.034
	NDA	H28–O12	0.43	1.090
		H29–O20	0.25	1.362
		H2–O20	0.09	1.769

Table 5 shows that H27 in DDA and NDA formed a bond with O24 on the surface of magnesite (101). For DDA and NDA, the population values were 0.19 and 0.17, and the bond lengths were 1.575 Å and 1.570 Å, respectively. From the perspective of population value and bond length, the bond formation between DDA and the surface of magnesite (101) was slightly stronger than that of NDA. This finding shows that DDA more easily adsorbs to magnesite in the absence of *n*-octanol.

Table 5 shows that H27 in DDA and NDA formed a bond with O34 on the surface of dolomite (101). For DDA and NDA, the population values were 0.19 and 0.23, respectively, and the bond lengths were 1.737 Å and 1.713 Å, respectively. From the perspective of population value and bond length, the bond between NDA and the surface of dolomite (101) was slightly stronger than that of DDA. This finding shows that NDA is more likely to adsorb to dolomite, which is consistent with the above test results.

Table 5 shows that H2 in DDA formed a bond with O20 on the surface of quartz (101), with a population value of 0.47 and a bond length of 1.034 Å. H28 and H2 of dodecylamine ions in NDA formed bonds with O12 and O20 on the quartz (101) surface, respectively, with population values of 0.43 and 0.09 and bond lengths of 1.090 Å and 1.769 Å, respectively. The H29 of *n*-octanol in NDA formed a bond with the O20 of the quartz (101) surface, with a population of 0.25 and a bond length of 1.362 Å. The adsorption sites of quartz increased, and the population value of bonds was relatively large, forming multiple H–O bonds, which markedly enhanced the adsorption of the agent on quartz. From the perspective of population value and bond length, the bond between NDA and the surface of quartz (101) was slightly stronger than that of DDA. This finding shows that NDA is more likely to adsorb to quartz, which is consistent with the above test results.

The above simulation results show that DDA can adsorb to magnesite, dolomite and quartz, and the adsorption effect was strongest on quartz. After the addition of *n*-octanol, the adsorption energies of the collectors to magnesite, dolomite and quartz all decreased. The population value of the collector bonding with dolomite and quartz increased, and the adsorption sites of quartz increased, whereas the population value of the collector bonding with magnesite decreased. This finding shows that the collector's interaction with dolomite and quartz was enhanced, whereas the change in the interaction intensity with magnesite was uncertain; the decrease in the adsorption energy indicated that the effect of the two was enhanced, whereas the bond population value indicated that the effect of the two was weakened. Therefore, the overall effect was uncertain.

## Conclusion

1. The test results show that increasing the dosage of DDA can increase the floating rate of magnesite, dolomite and quartz. When the dosage exceeded 50 mg/L, the flotation rate of quartz can exceeded 95%, whereas the flotation rates of magnesite and dolomite were less than 60%. After adding *n*-octanol, the floating rate of magnesite decreased, the floating rates of dolomite and quartz increased, and a lower dosage of DDA was required for floating rates of quartz exceeding 90%.
2. The simulation results show that the adsorption energies of DDA to the surface of magnesite (101), dolomite (101) and quartz (101) were − 197.14 kJ/mol, − 127.96 kJ/mol, and − 464.13 kJ/mol, respectively. Charge analysis showed that the hydrogen atoms on DDA and the oxygen atoms on the surface of magnesite, dolomite and quartz were partially transferred to each other. Moreover, the hydrogen atoms in DDA formed bonds with the oxygen atoms in magnesite, dolomite and quartz, indicating that DDA can adsorb to magnesite, dolomite and quartz, and this affect was strongest for quartz. After the addition of *n*-octanol, the adsorption energies of the collectors to magnesite, dolomite and quartz all decreased. The adsorption energies of NDA on the surfaces of magnesite (101), dolomite (101) and quartz (101) were − 330.90 kJ/mol, − 206.66 kJ/mol and − 573.14 kJ/mol, respectively. Charge analysis showed that the hydrogen atoms on the NDA and the oxygen atoms on the surface of magnesite, dolomite and quartz were partially transferred to each other. The population value of the collector bonding with dolomite and quartz and the adsorption sites of quartz increased, whereas the population value of the collector bonding with magnesite decreased. This finding shows that the collector's interaction with dolomite and quartz was enhanced, whereas the change in the interaction intensity with magnesite was uncertain. The flotation test results show that the floating rate of dolomite and quartz increased after adding *n*-octanol, which is consistent with the simulation results. The floating rate of magnesite was reduced, which may be due to the weakening of the interaction between the collector and magnesite, mineral dissolution or the influence of coexisting ions.
3. The results of the experiment and simulation show that in the presence of *n*-octanol, the dosage of chemicals can be reduced, and a good impurity removal effect can be achieved. When removing silicon from magnesite ore by reverse flotation, part of the calcium can be removed, and the purity of magnesite can be improved.

## Data Availability

All data generated or analysed during this study are included in this manuscript.
